# 5-Azacitidine Induces Cell Death in a Tissue Culture of *Brachypodium distachyon*

**DOI:** 10.3390/ijms19061806

**Published:** 2018-06-19

**Authors:** Alexander Betekhtin, Anna Milewska-Hendel, Lukasz Chajec, Magdalena Rojek, Katarzyna Nowak, Jolanta Kwasniewska, Elzbieta Wolny, Ewa Kurczynska, Robert Hasterok

**Affiliations:** 1 Department of Plant Anatomy and Cytology, Faculty of Biology and Environmental Protection, University of Silesia in Katowice, 40-032 Katowice, Poland; magdalena.rojek@us.edu.pl (M.R.); jolanta.kwasniewska@us.edu.pl (J.K.); elzbieta.wolny@us.edu.pl (E.W.); robert.hasterok@us.edu.pl (R.H.); 2 Department of Cell Biology, Faculty of Biology and Environmental Protection, University of Silesia in Katowice, 40-032 Katowice, Poland; anna.milewska@us.edu.pl (A.M.-H.); ewa.kurczynska@us.edu.pl (E.K.); 3 Department of Animal Histology and Embryology, Faculty of Biology and Environmental Protection, University of Silesia in Katowice, 40-032 Katowice, Poland; lukasz.chajec@us.edu.pl; 4 Department of Genetics, Faculty of Biology and Environmental Protection, University of Silesia in Katowice, 40-032 Katowice, Poland; katarzyna.nowak@us.edu.pl

**Keywords:** Brachypodium, 5-azacitidine, cell death, tissue culture

## Abstract

Morphological and histological observations revealed that, at a concentration of 50 µM, 5-azacitidine (5-azaC) totally inhibited the induction of embryogenic masses (EM), while the cultivation of explants (zygotic embryos; ZEs) in the presence of 5 µM of 5-azaC led to the formation of a callus with EM in 10% of the cases. Transmission electron microscopy (TEM) analyzes revealed the presence of the morphological and ultrastructural features that are typical for the vacuolar type of cell death in the callus cells that were treated. A TUNEL assay confirmed the presence of DNA double-strand breaks for the callus cells that had been treated with both 5 and 50 µM 5-azaC concentrations. Analysis of the gene expression of selected cell death markers demonstrated a reduced expression of metacaspase, protein executer 1 (EX1), and thioredoxin (TRX) in the callus cells that had been treated compared to the control culture. The strongest increase in the gene activity was characteristic for glutathione S-transferase (GST). Our studies also included an analysis of the distribution of some arabinogalactan proteins (AGPs) and extensin epitopes, which can be used as markers of cells that are undergoing death in a *Brachypodium distachyon* tissue culture.

## 1. Introduction

*Brachypodium distachyon* (Brachypodium) has become a useful model system to study various aspects of a tissue culture in grasses. To date, it has been used to analyze the chemical composition of the cell wall of different organs, tissues, and cell types at different stages of development [1,2]; the functions of different genes [3]; and the genetic regulation of somatic embryogenesis (SE) in grass species [4]. 

Plant cells that are introduced into a tissue culture undergo successive changes, which include dedifferentiation, the acquisition of pluripotency or totipotency, the proliferation of cells, differentiation, and finally the formation of embryos [5]. DNA methylation plays a pivotal role during the processes of dedifferentiation/differentiation and in the formation of the embryogenic masses. This DNA modification regulates gene activity in a positive or negative manner [6]. Although in most cases it causes gene inactivation [7,8], it has also been demonstrated that the methylation of individual genes may also induce their expression [9]. The DNA-hypomethylating agent, 5-azacitidine (5-azaC), is often used to reveal the functions of DNA dynamics. Yamamoto et al. [10] showed that the inhibition of DNA methylation by 5-azaC in a carrot culture impeded the formation of embryogenic cell clumps from the carrot epidermal cells. The impact of DNA methylation on somatic embryogenesis (SE) in *Arabidopsis thaliana* (Arabidopsis) demonstrated a decrease in the global DNA methylation level during SE that accompanied with the upregulation of DNA methylases and the downregulation of DNA demethylases [11]. These authors revealed significant repression of *LEC2*, *LEC1*, and *BBM* in vitro under the treatment of 5-azaC. As a consequence, this culture was incapable for SE induction. The research on *Theobroma cacao* demonstrated that the long-term SE induced the decline in embryogenic potential, which can be reversible by the addition of 5-azaC [11]. The experimental changes in the total DNA methylation level may play a critical role in the different processes of plant development. For example, treatment of cold-sensitive Arabidopsis plants with 5-azaC makes the process of vernalisation unnecessary [12]. It was demonstrated for barley coleoptiles that at a concentration of 100 µg/mL, 5-azaC may have an effect on the stimulation of DNA fragmentation [13]. Transcriptome analyzes of Arabidopsis seedlings treated with either 5-azaC or zebularine revealed a significant number of upregulated genes especially linked with transposable element genes. This showed that these two agents have a disproportionately large influence on the loci that are silenced by DNA methylation [14].

DNA cleavage and the expression of specific genes—for example genes encoding for metacaspase, glutathione S-transferase, and protein executer 1—are specific features of programmed cell death [15,16,17,18]. This process is also characterized by the activation of endonucleases, which produce single-strand breaks and cause low molecular weight DNA (mono- and oligonucleosomes) fragments to be formed [15]. One of the methods that has been described to identify this process is TdT-mediated dUTP nick end labelling, which is known as a TUNEL assay [19].

Hydroxyproline-rich glycoproteins (HRGPs) are important plant cell wall components that are involved in many aspects of plant growth and development, including the responses to stress [20,21]. The HRGP superfamily comprises a continuum of molecules from the nonglycosylated/minimally glycosylated proline-rich proteins (PRPs) to the moderately glycosylated extensins to the highly glycosylated AGPs (for review, see [22,23]). Based on literature data, it is hypothesized that AGPs and extensins may be involved in the cell death process [24].

The aim of this study was to determine the effects of 5-azaC during the formation of the embryogenic masses as well as to characterize the morphology, histology, ultrastructure, and chemical composition of selected components of the cell wall. The expression of selected genes in both the experimental (treated with 5-azaC) and control explants was also determined.

## 2. Results

### 2.1. Morphological, Histological, Cytological, and Ultrastructural Observations in the Brachypodium Callus

**Figure 1:**
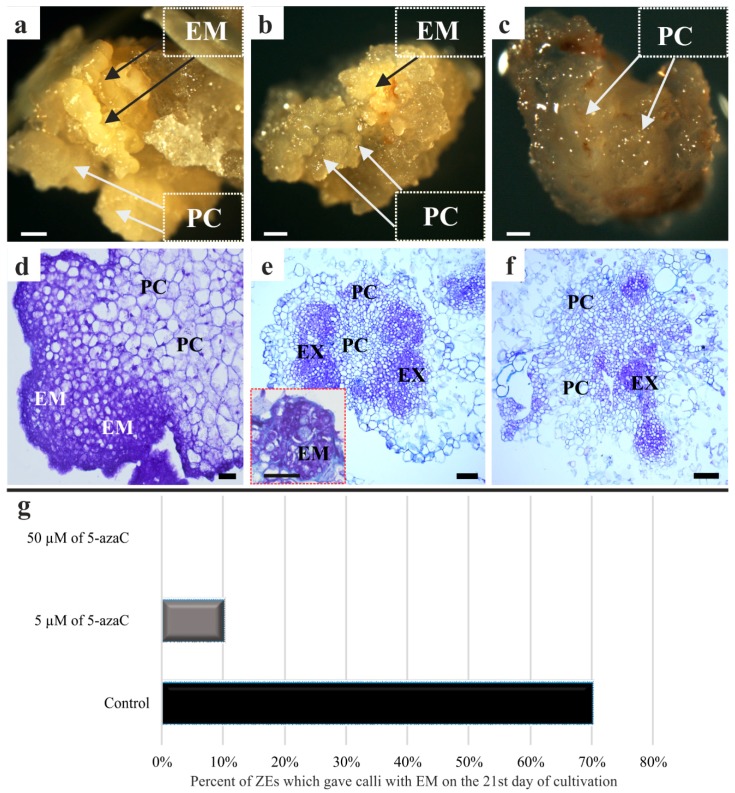
Morphology (**a**–**c**) and histology (**d**–**f**) of the Brachypodium callus. (**a**,**d**) Control callus; (**b**,**e**) callus that has been treated with 5 µM of 5-azaC, (inset figure on (**e**) demonstrated embryogenic masses (EM)) and (**c**,**f**) callus that has been treated with 50 µM of 5-azaC (black arrows: EM; white arrows; parenchymatous cells: PC; cells of an explant: EX). (**g**) frequency of ZEs which gave calli with EM on the 21st day of cultivation. Scale bars, (**a**–**c**) 200 µm; (**d**) 20 µm; (**e**,**f**) = 100 µm.

In a previous work, we demonstrated that the embryogenic callus of Brachypodium can be successfully obtained from immature embryos [2]. The results presented here show that after a week of cultivation on a callus induction medium (CIM), neither the explants in the control conditions with a developing callus nor those that had been grown in the presence of 5 and 50 µM of 5-azaC displayed any morphological differences. These, however, became clearly visible after three weeks of cultivation (Figure 1a–c). The Brachypodium embryogenic callus of the control explants (Figure 1a,d) and the explants that had been treated with 5 µM of 5-azaC (Figure 1b,e) were primarily composed of two different types of callus areas: (i) one that is vitreous and friable, which contained parenchymatous cells (PC) and (ii) a compact callus that was represented by embryogenic masses (EM) that were yellowish in color. After 21 days of cultivation, 70% of the control callus and 10% of callus grown in the presence of 5 µM 5-azaC had formed EM (Figure 1g). The treatment with 50 µM of 5-azaC led only to the formation of a non-embryogenic callus, which was composed of a PC with no visible EM (Figure 1c,f). The histological sections clearly demonstrated that apart from normal embryogenic cells, highly vacuolated PC were also present (Figure 1d–f). 

**Figure 2:**
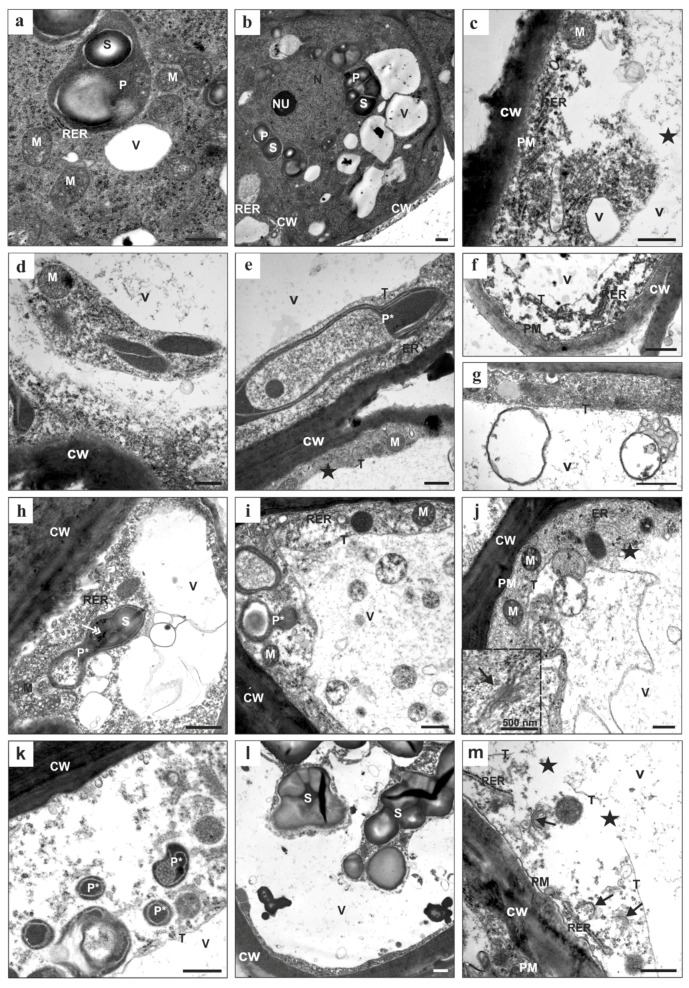
Callus cell ultrastructure in the control (**a**,**b**) and in the callus that has been treated with 5 µM (**c**–**f**) and 50 µM (**g**–**m**) of 5-azaC. CW: cell wall; ER: endoplasmic reticulum; M: mitochondria; N: nucleus; NU: nucleolus; P: plastid; P*: plastid with an altered ultrastructure; PM: plasma membrane; RER: rough endoplasmic reticulum; S: starch; T: tonoplast; V: vacuole; arrows: dictyosomes of Golgi apparatus; double arrow: plastoglobules; asterisks: tonoplast breakdown. Scale bars, 1 µm.

Transmission electron microscopy (TEM) analysis revealed that regardless of the duration of the culture, the cytoplasm of the explant cells of the control callus was electron dense and numerous organelles such as a prominent nucleus (N) with a nucleolus (NU), mitochondria (M), plastids with numerous starch grains (S), an endoplasmic reticulum (ER), and small vacuoles (V) were clearly visible (Figure 2a,b). The plastids (P) with starch (S) were located in the vicinity of the nucleus and the cytoplasm contained numerous ribosomes. The cells were characterized by the presence of thin cell walls, which may indicate that cytokinesis occurred during the cultivation of the tissue culture.

In the cells from the callus that had been treated with 5-azaC, similar and prominent changes in the cytoplasm of the parenchymatous cells were detected regardless of the concentration of this hypomethylating agent or the duration of the culture (Figure 2c–m). These included the fragmentation of the ER followed by the total disintegration of this organelle. Disintegration was also observed in the case of the dictyosomes of the Golgi apparatus (GA; Figure 2m, arrows). Both the ER cisterns and the GA dictyosomes became less pronounced and had no well-defined shape, which may indicate that their membranes were disintegrating (Figure 2j; inset). Prominent changes in the plastid ultrastructure were also evident (Figure 2e,h,i,k,l). Large and numerous starch grains were present in the amyloplasts. The density of the plastid stroma and the number of plastoglobuli increased (Figure 2h, double arrow), which was accompanied by the disintegration of the internal membrane (Figure 2e,k). The plastids that were fused to the vacuole appeared to rupture and this process was associated with the degradation of their outer membrane (Figure 2j,l). They disintegrated much earlier compared to the mitochondria, which were preserved for the longest period of time compared to the other organelles (Figure 2c,e,i). Although the collapse of the tonoplast was the most prominent feature in all of the cells that were investigated (Figure 2c,j,m; asterisk), the progress of the interruption of the tonoplast membrane was gradual. Numerous membrane-bound bodies with remnants of the cytoplasm present in most of them were observed inside the vacuolar compartments (Figure 2g,i,j). The plasma membrane was intact almost until the end of cell death. As cell death proceeded, the cytoplasm narrowed (Figure 2e,l), was located around the periphery of a cell, and became more translucent (Figure 2c,k,m). Numerous electron-lucent areas were detected within the cytoplasm. Inside the area of the former vacuole granular material, parts of the cytoplasm with plastids and other organelles in a state of decay were present (Figure 2d,l). In general, during cell death, the tonoplast disruption preceded the breakdown of the plasma membrane and it appears that the collapse of the tonoplast is the main executor of cell death. 

**Figure 3:**
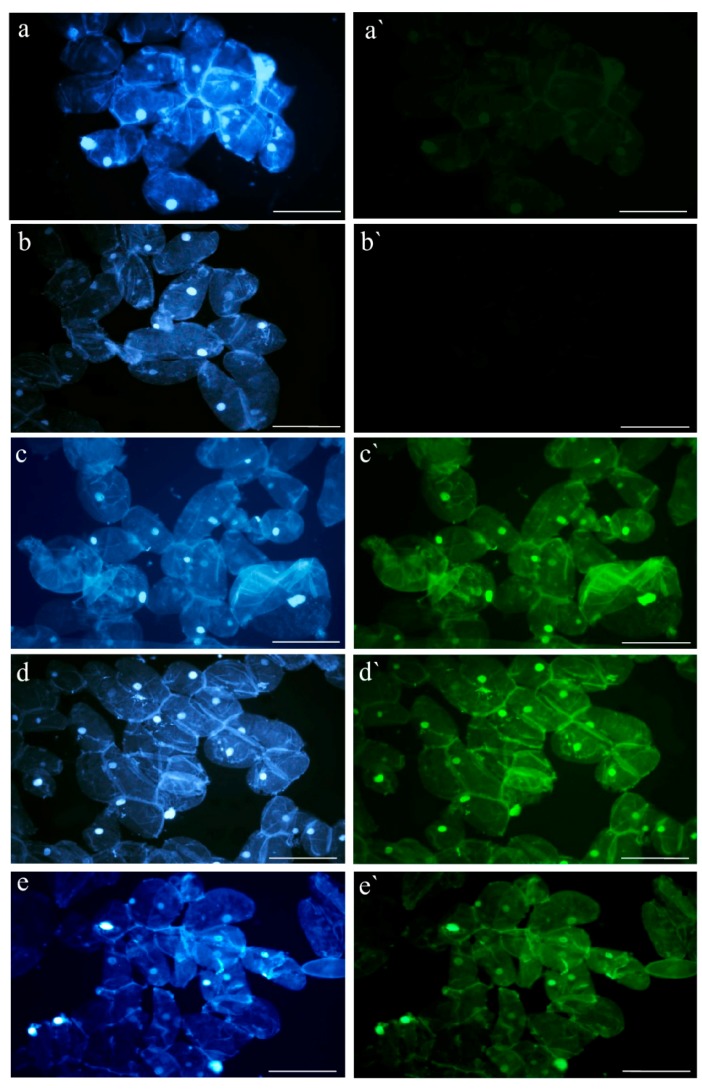
In situ detection of DNA fragmentation in the Brachypodium callus cells on the 14th day of the culture using the TUNEL assay. Blue fluorescence: DAPI staining (**a**–**e**), green fluorescence: FITC marking TUNEL-positive nuclei (**a`**–**e`**). (**a**,**a`**) control callus; (**b**,**b`**) negative control in TUNEL reaction; (**c**,**c`**) positive control in TUNEL reaction; (**d**,**d`**) callus that has been treated with 5 μM of 5-azaC; (**e**,**e`**) callus that has been treated with 50 μM of 5-azaC. Scale bars, 50 µm.

Application of the TUNEL assay permitted the DNA fragmentation in the Brachypodium callus nuclei to be visualized. Although this fragmentation was detected in the control callus cells and the callus cells that had been treated with 5 or 50 μM of 5-azaC (Figure 3), only weak fluorescence was observed in the control cells, whereas strong FITC signals were observed in the callus cells that had been treated with the hypomethylating agent. The frequencies of labelled nuclei did not change during the 21 days of the in vitro culture. TUNEL-specific fluorescence was not observed in the negative control (no terminal transferase was used; Figure 3b,b`), whereas all of the nuclei were labeled in the positive control (Figure 3c,c`). No differences in the frequencies of the TUNEL-positive nuclei were observed on the 7th, 14th, and 21st days of the culture (Figure 3; only the results for the 14th day of the in vitro culture are shown).

### 2.2. RT-PCR

**Figure 4:**
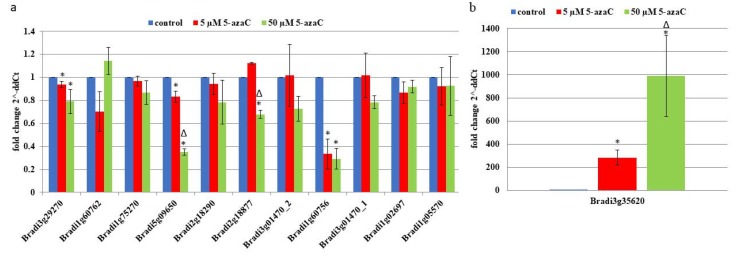
Relative expression levels of the selected PCD-related genes (**a**), in the explants that were cultivated on the CIM medium supplemented with 5 and 50 μM of 5-azaC (**b**). Relative expression levels were normalized to an internal control (AK437296, gene encoding for ubiquitin) and calibrated to the control culture (CIM medium with no 5-azaC). *: value is significantly different from the control culture (*p* < 0.05; *n* = 3 ± SD), Δ: value is significantly different from the 5 μM of 5-azaC-treated culture (*p* < 0.05; *n* = 3 ± SD).

Complementary DNAs (cDNAs) were used as templates for the PCR amplification with the appropriate primers (Table 1). In order to test the hypothesis of whether 5-azaC-induced cell death is programmed or not, 12 genes were assessed. Almost all of them were characterized by a decrease in their expression after treatment with this hypomethylating agent. The lowest expression levels, whose values were significantly different that the control in the culture that had been treated with 5 and 50 µM of 5-azaC, were these for the genes Bradi1g60756.1, Bradi3g29270 and Bradi5g09650.1, which encode for metacaspase, protein executer 1 (EX1) and thioredoxin (TRX), respectively (Figure 4a). Values that were significantly different between only the 5 and 50 μM of the 5-azaC-treated cultures were demonstrated for the genes Bradi1g60756.1 (metacaspase) and Bradi2g18877.1 (hexokinase). The strongest increase in gene activity was characteristic for Bradi3g35620.1, which encodes for glutathione S-transferase (GST) and it was up to 300- and 1000-fold higher for the 5 and 50 µM 5-azaC-treated cultures, respectively (Figure 4b). 

### 2.3. AGPs and Extensins under Different 5-azaC Treatments

**Figure 5:**
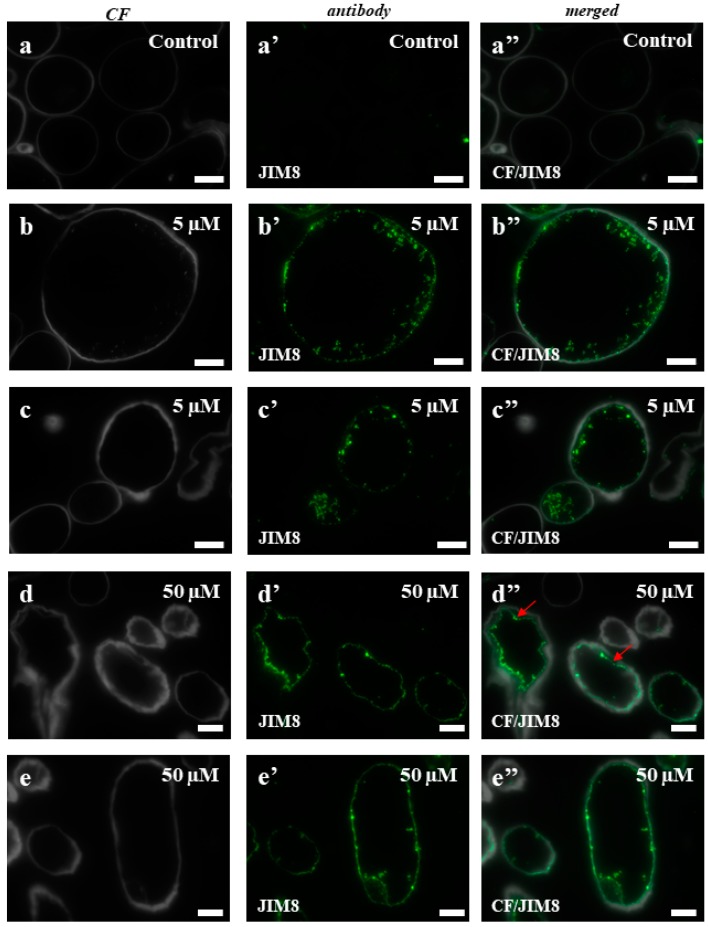
Immunolocalization of JIM8 in the Brachypodium callus. (**a**–**a”**): control callus; (**b**–**b”**,**c**–**c”**): callus that has been treated with 5 µM of 5-azaC. (**d**–**d”**,**e**–**e”**): callus that has been treated with 50 µM of 5-azaC. (**d”**): red arrows demonstrate the detachment of the plasmalemma from the cell wall. Scale bars, 10 µm.

**Figure 6:**
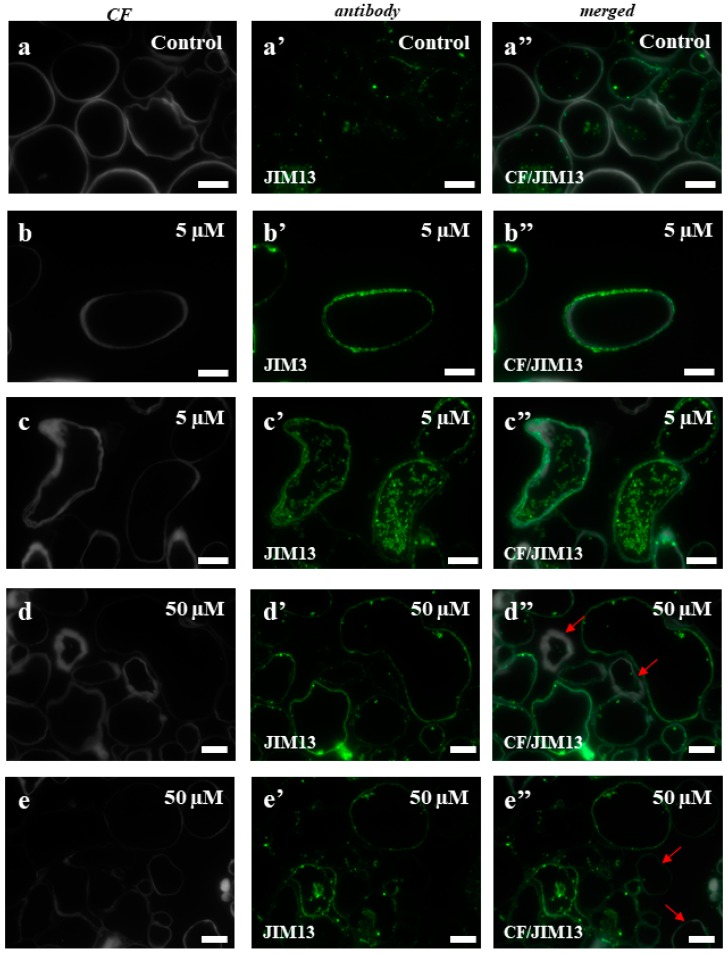
Immunolocalization of JIM13 in the Brachypodium callus. (**a**–**a”**): control callus. (**b**–**b”**,**c**–**c”**): callus that has been treated with 5 µM of 5-azaC. (**d**–**d”**,**e**–**e”**): callus that has been treated with 50 µM of 5-azaC. The cells with a total absence of the signals are indicated by red arrows. Scale bars, 10 µm.

**Figure A1:**
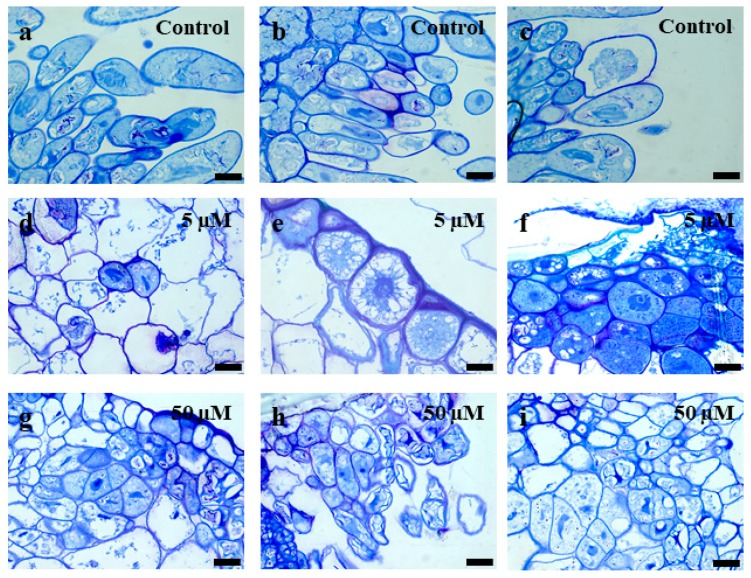
Histological sections of the Brachypodium callus stained with a 0.05% (*w*/*v*) toluidine blue O. (**a**–**c**): control callus; (**d**–**f**): callus that has been treated with 5 µM of 5-azaC. (**g**–**i**): callus that has been treated with 50 µM of 5-azaC. Scale bars, 10 µm.

To test whether the selected AGP and extensin epitopes correlated with the cytological/ultrastructural changes that accompany the cell death, immunocytochemistry experiments using selected monoclonal antibodies were performed. The histological sections of the cell types that were subjected to immunocytochemical analyzes are presented in Figure A1. Two epitopes (JIM8, JIM13) for AGP and three (LM1, LM11, JIM12) for extensins were used in this analysis. Because it is difficult to identify the separate fluorescence signals in the cell wall and plasma membrane using light microscopy, in the case of the AGP epitopes, these two compartments are described together. The signals inside the cells were localized in the intracellular compartments. The immunolocalisation of JIM8 showed almost a total absence of a fluorescence signal in any part of the control callus cells (Figure 5a–a”), which contrasted with a strong signal in the plasmalemma and intracellular compartment of cells from the material that had been treated with 5 and 50 µM of 5-azaC (Figure 5b–b”,c–c”,d–d”,e–e”). The plasmolysis of some of the cells that had been treated with the higher concentration of the hypomethylating agent confirmed the fact that the localization of this epitope is in plasmalemma (Figure 5d”, red arrows). In the control callus of Brachypodium, a patchy distribution of JIM13 in the cell wall and cytoplasmic compartments was observed (Figure 6a–a”). The callus that had been treated with 5 µM of 5-azaC was characterized by the presence of this epitope on the cell wall and intracellular compartments (Figure 6b–b”,c–c”). In contrast, the callus that had been treated with 50 µM of 5-azaC expressed the JIM13 signals in the cell wall and also had some weak signals in the intracellular compartments (Figure 6d–d”,e–e”). Some of the cells were characterized by a total absence of signals (Figure 6d”,e”, red arrows).

**Figure 7:**
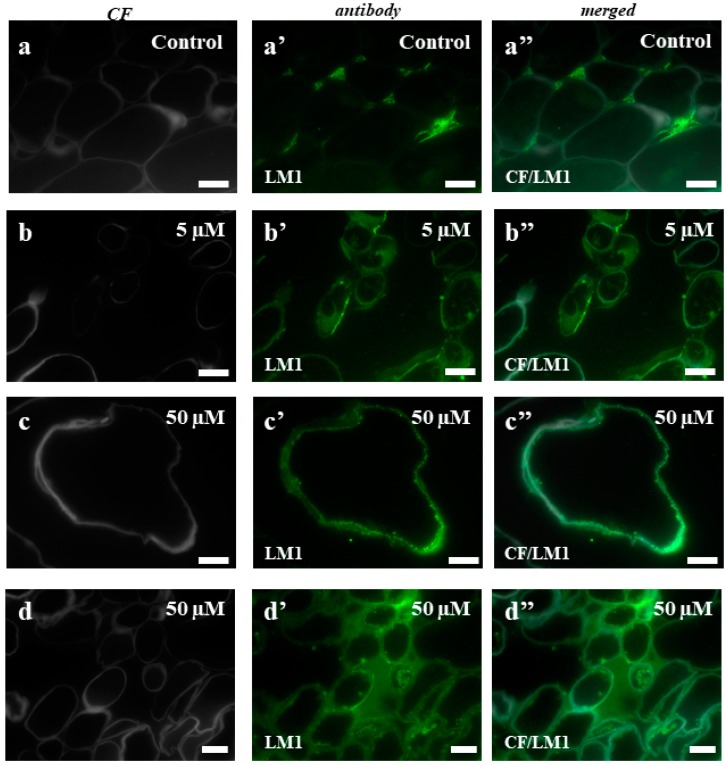
Immunolocalization of LM1 in the Brachypodium callus. (**a**–**a”**): control callus. (**b**–**b”**): callus that has been treated with 5 µM of 5-azaC. (**c**–**c”**,**d**–**d”**): callus that has been treated with 50 µM of 5-azaC. The greenish background on these photomicrographs is due to the autofluorescence. Scale bars, 10 µm.

**Figure 8:**
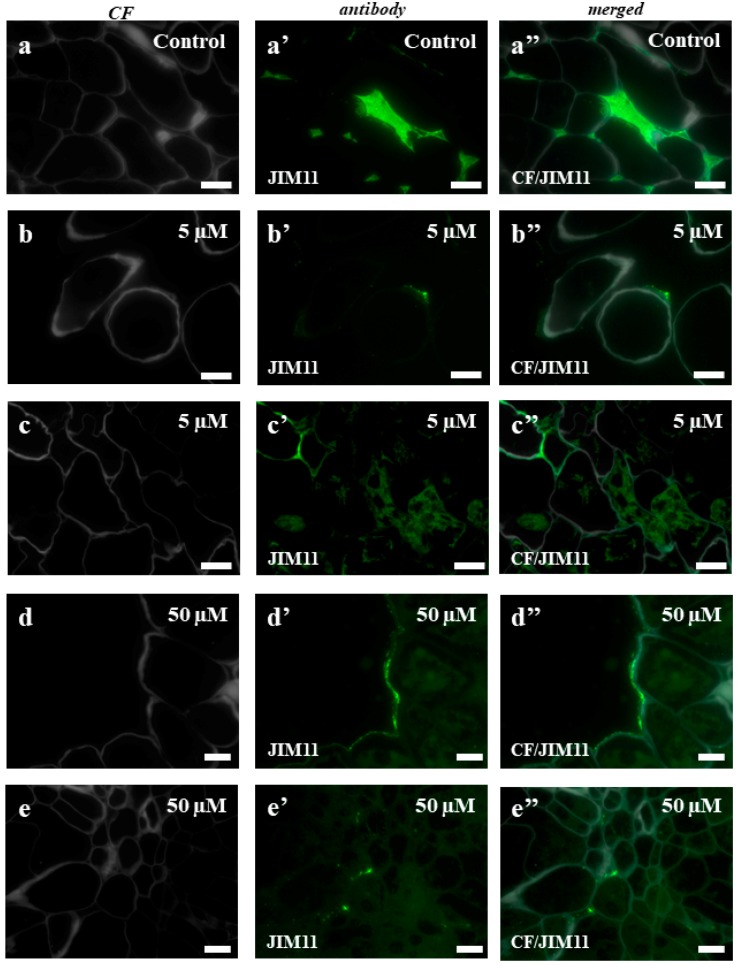
Immunolocalization of JIM11 in the Brachypodium callus. (**a**–**a”**): control callus. (**b**–**b”**,**c**–**c”**): callus that has been treated with 5 µM of 5-azaC. (**d**–**d”**,**e**–**e”**): callus that has been treated with 50 µM of 5-azaC. The greenish background on these photomicrographs is due to the autofluorescence. Scale bars, 10 µm.

**Figure 9:**
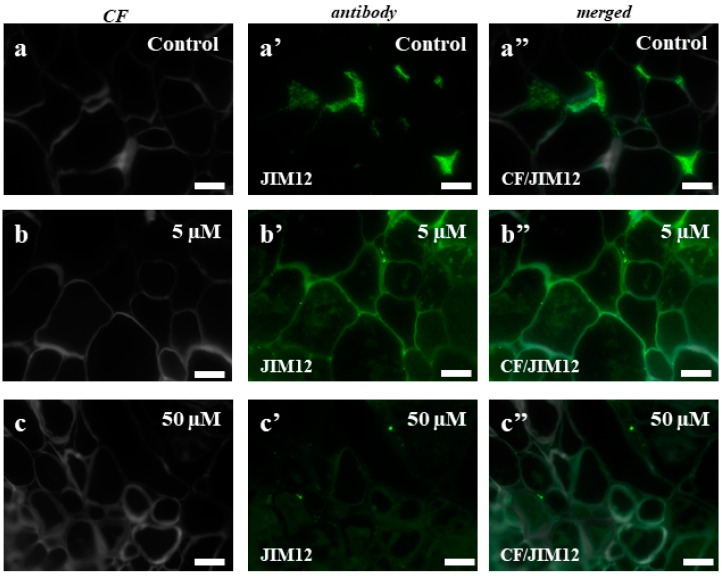
Immunolocalization of JIM12 in the Brachypodium callus. (**a**–**a”**): control callus. (**b**–**b”**): callus that has been treated with 5 µM of 5-azaC. (**c**–**c”**) callus that has been treated with 50 µM of 5-azaC. The greenish background on these photomicrographs is due to the autofluorescence. Scale bars, 10 µm.

In the case of the control callus, the experiments with the LM1 antibody showed the localization of this epitope in the intercellular spaces (Figure 7a–a”). The callus that had been treated with 5 µM of 5-azaC displayed the presence of this epitope on the surface of some cells (Figure 7b–b”), which contrasted with the intracellular localization of the signals from this antibody in the callus after the treatment with 50 µM of 5-azaC (Figure 7c–c”,d–d”). In the control, JIM11 only had signals in the intercellular spaces (Figure 8a–a”). The immunolocalization of JIM11 in the experimental material demonstrated the presence of weak signals in both the callus cells that had been treated with 5 µM and 50 µM of 5-azaC (Figure 8b–b”,c–c”,d–d”,e–e”). These signals, however, were found only outside the cell wall and in the cell wall of some callus cells. Interestingly, the JIM12 extensin epitope had an intercellular localization in both the treated and control calli (Figure 9). However, in the callus that had been treated with both concentrations of 5-azaC, the signals of this antibody were almost absent (Figure 9b–b”,c–c”). 

## 3. Discussion

In higher plants, DNA is strongly methylated on the cytosine residues. 5-azaC is a chemical analogue of cytosine that can be incorporated into the DNA structure during its replication, thereby replacing the cytosine residues therein. Such a substitution makes DNA methylation via the formation of 5-methylcytosines impossible. It has also been shown that the 5-azaC-induced DNA hypomethylation can restore transgene expression in long-term cultivated cell cultures of birch [25] and that changes in DNA-methylation can be induced under stress conditions [26,27,28]. In plants, the effect of the application 5-azaC appears to be species dependent. Solis et al. [29] demonstrated that it may promote the initiation of microspore embryogenesis by decreasing global DNA methylation, while a double treatment using 2,4-D and 5-azaC improved somatic embryo induction in a tissue culture of *Acca sellowiana* [30]. It was also revealed that treatment with 5-azaC caused both callus induction and somatic embryogenesis in *Fraxinus mandshurica* [31]. 

The results of our morphological and histological observations suggest that the fact that global DNA methylation was affected by 5-azaC does not play a pivotal role in the processes of callus formation. However, it does appear to be crucial for the formation of the EM. It is known that treatment with 5-azaC induces cell death in some cereals, for example in barley cells from the first leaf display an apoptotic pathway of cell death. The inhibition of nuclear DNA fragmentation during the development of the first leaf cells and an increase in the amount of DNA was also demonstrated in barley cells [32]. A similar way of cell death was demonstrated in the coleoptile and initial leaf seedlings during the early stages of wheat development [6,33]. 

It should be taken into account that 5-azaC can have dose-dependent secondary effects and cell toxicity mediated by covalent trapping of DNA methyltransferase rather than by DNA demethylation itself [10,34,35,36,37]. In Arabidopsis and *Thlaspi arvense*, flowering time was advanced at 250 µM of 5-azaC while higher concentrations were inhibitory to bolting and flower development [38]. It was demonstrated in *Sorghum bicolor* that 5-azaC concentration used for seedlings (310 µM) proved to be toxic to calli [39]. In hybrid larch, treatment with 100 µM of 5-azaC led to a dramatic decrease in the number of embryonal masses as well as embryogenic potential, which was attributed to 5-azaC toxicity [36]. To sum up, the decrease/absence of EMs on the surface of Brachypodium callus treated with 5-azaC may be due to the cytotoxic effect through the damage of DNA leading to cell death. 

The ultrastructural changes within the cells that were observed during the culture with 5-azaC may indicate that the process of cell death is not necrosis. We propose that it may have the features of a programmed process. The traits described for cell death that were observed in this study indicate that it may be a vacuolar cell death, which is manifested by a gradual decrease in the volume of cytoplasm and a significant increase in the vacuole volume as well as volume presence of the cytoplasm content of the cells inside the vacuole compartment and the subsequent degradation of the cargo [40]. This type of cell death was also described during embryo development as well as during tissue and organ differentiation, which is either a normal part of the plant developmental program or a response to stressful conditions [41,42,43,44,45]. Interestingly, in our research, we observed the increasing number of plastoglobuli, which may be linked with the upregulation of plastid lipid metabolism in response to oxidative stress provoked by 5-azaC. It was demonstrated that the number of plastoglobules increases in plants subjected to environmental conditions—such as drought, high saline concentrations, viral infections, and increased ozone concentration—which increases the oxidative stress on the photosynthetic apparatus [46,47,48,49]. 

The TUNEL assay is widely used to confirm the presence of double-strand breaks (DSBs) [19,50,51]. Using this approach, it was demonstrated that the surface cells covering the somatic embryos of *Daucus carota* underwent cell death before peeling off [52]. It was also successfully applied to demonstrate cell death after the addition of the Yariv reagent to Arabidopsis cell cultures [24]. In our research, use of the TUNEL assay demonstrated the presence of TUNEL-positive cells on the seventh day of cultivation and they were represented by almost 100% of the cells, which could indicate the presence of cell death. It should be noted that TUNEL-positive control nuclei were observed at a frequency of 8.7%, thus confirming the well-known fact that DSBs can be generated spontaneously [6].

As we found almost all of the TUNEL-positive cells on the seventh day of cultivation, we chose this day to analyze the particular expression of the genes that participate in cell death in Brachypodium callus using RT-PCR. As was mentioned earlier, the strongest expression was characteristic for GSTs, which are ubiquitous enzymes that catalyze the conjugation of toxic xenobiotics and oxidatively produced compounds to reduced glutathione, which facilitates the metabolism of toxic substances, sequestration, or removal [53]. The importance of this family of enzymes is confirmed by their abundance in a cell, since they contribute to at least 1% of the total amount of cell proteins [17]. The genome of Arabidopsis contains 48 GST-like genes [54]. The influence of adverse biotic and abiotic factors on the cells induce the expression of GST genes [18,55]. Such factors may include dehydration, herbicides, damage, cold, hypoxia, H_2_O_2_, UV, phosphate starvation, metal ions, high salt concentrations, and pathogens. It should be noted that the activity of GST may be also connected with TRX and that particular TRXs may also be reduced via the glutathione/glutaredoxin system [56]. In our experiments on the Brachypodium callus, we observed a decrease in the TRX expression. Cultivation of the callus in the dark together with the 5-azaC treatment could lead to the inactivation of TRXs. The importance of light for TRX activation was demonstrated for the potato plastidic enzymes, which are inactive due to the oxidized state of their critical cysteinyl residues in the dark [57].

Metacaspase seems to be very important in determining the mode of cell death as was demonstrated for vacuolar death in the embryo suspensor of Norway spruce, which required autophagy [58]. The activation of autophagy was connected with the downstream signaling of metacaspase mcII-Pa. These authors highlighted that the genetic suppression of the metacaspase-autophagy pathway induced a switch from a vacuolar to a necrotic cell death. In the present work, we show a decrease in the expression on the metacaspase, which corroborates the results of TEM analyzes, where we observed the typical features of the vacuolar cell death. A decrease in the expression of EX1 was also observed in this work. EX1 and EX2 are plastid proteins and their inactivation in the *flu* mutant of Arabidopsis led to the loss of chloroplast integrity that precedes the rupture of the central vacuole and the final collapse of a cell [59]. The expression levels of both EX1 and EX2 increase during biotic and abiotic stress in wild type plants. Thus, the chloroplasts may serve as sensors of the environmental changes that activate a broad range of stress responses [16]. A decrease of the expression level of EX1 in the Brachypodium callus appears to be connected with the absence of a developed chloroplast system. Since the Brachypodium callus was cultivated in the dark, its cells only contained proplastids that were deprived of a prolamellar body along with some loosely associated membranes (Figure 2h,i), which may be one of the reasons for the EX1 inactivation. Another rationale might be the accumulation of reactive oxygen species after cultivation with 5-azaC, which leads to a decrease in the EX1 expression. 

The epitopes of AGPs and extensins were selected for the studies presented here because of their known role in different developmental processes [20,21,60,61], including somatic embryogenesis [61,62,63] and cell death [24,64]. Our results showed that the distribution of these AGP and extensin epitopes differed in the treated callus compared to the control explants and that the reaction to 5-azaC was dose dependent. Moreover, all of the detected changes in the presence of the tested epitopes were typical for the cells, which exhibited features on the cellular and ultrastructural level indicate that they undergo death other than necrosis, and as we postulate, it is a vacuolar cell death. AGPs can be considered to be molecular markers of cell death during microsporogenesis in Arabidopsis, where it was revealed that the tapetum cells show the morphological features that are typical for programmed cell death and have the high presence of AGPs recognized by JIM8 and JIM13 during their degeneration [65]. It has also been documented that the epitopes of AGPs are recognized by the JIM13 and JIM14 antibodies, which are associated with cells that are committed to programmed cell death [66]. In our work, we used two monoclonal antibodies, JIM8 and JIM13, that recognize the AGP epitopes and both of these showed the strongest signals in the callus that had been treated with 5-azaC and were almost absent in the control callus. These results may suggest that in the Brachypodium callus that had been treated with 5-azaC, the cells that are “decorated” by these signals undergo cell death, and if so, these epitopes may serve as the molecular markers of cell death. 

Unlike AGPs, the immunodetection of extensins in the Brachypodium explants demonstrated a decrease in the fluorescence signals of each epitope in the callus that had been treated with 5-azaC that was analyzed. In the cell walls of bean cell suspensions during habituation and dehabituation to dichlobenil, the HPRG epitope that is recognized by LM1 was detected throughout the cell walls of the non-habituated cell clusters and the epitope appeared mainly in the cell wall corners and on the outer surfaces of the walls in the habituated cells [67]. A similar distribution of this epitope was detected in the Brachypodium callus that had been treated with 5-azaC. During the somatic embryogenesis of banana, it was shown that the HRGP that is recognized by JIM11 was present in a very small amount in the nonembryogenic part of explant, but that a moderate fluorescence signal was found only at the surface of the cell aggregates [61]. The same studies showed that the JIM11 epitope was abundant in the cell walls and especially in the tricellular junctions of the inner cortical cells. A similar distribution was found in our study on Brachypodium. 

Based on these results, the main function of extensins in the Brachypodium callus is postulated to be ‘fixing’ the cells among each other as we found strong fluorescence signals in the intracellular spaces and their almost total absence in the callus that had been treated with 5-azaC. As was demonstrated earlier, during the final stages of cell death, the collapse of the cell walls into crushed layers requires their structural reorganization [65]. An extensin scaffold appears to be one of the most important components that is required for the self-assembly of the plant cell wall [68]. Research on an Arabidopsis mutant with a lethal knockout of a single extensin gene (*AtEXT3*) designated root-, shoot-, and hypocotyl-defective during embryogenesis was typified by an incomplete cross wall assembly [69]. These authors found that a defective wall assembly is a direct consequence of the absence of AtEXT3 and concluded that a protein containing hydroxyproline is an integral part of the cell wall of actively growing cells. The entrance of cells into the pathway that leads to death is accompanied by changes in the epitopes of AGPs and extensins that were analyzed. 

## 4. Materials and Methods

### 4.1. Plant Material Growth, In Vitro Culture Conditions, and 5-azaC Treatment

The embryogenic callus was obtained according to Betekhtin et al. [2]. Zygotic embryos (ZEs) were cultivated on a callus induction medium (CIM) that had been supplemented with 5 and 50 µM of 5-azaC as well as on a CIM medium with no 5-azaC (control). For each experiment, 100 ZEs were used. One passage of cultivation took 21 days and the material was collected and analyzed on the 7th, 14th, and 21st days of cultivation.

### 4.2. Histological Procedures

The procedures for embedding tissues in Steedman’s wax [70] and preparing the slides were done according to Wolny et al. [71]. For the toluidine blue staining, slides with tissue sections were de-embedded three times for 10 min in 99.8% ethanol and rehydrated in ethanol/distilled water for 10 min at each step (90%, 70%, 50%, 30% *v*/*v*, distilled water). The slides were then placed in an aqueous 0.01% toluidine blue solution for 10 min and rinsed three times in distilled water for 5 min each. The stained slides were then air dried and embedded in a mounting medium (DPX, Sigma-Aldrich, St. Louis, MO, USA). Images of the stained tissue sections were obtained using an Axio Imager Z2 microscope equipped with an AxioCam camera (Zeiss, Oberkochen, Germany).

### 4.3. TEM 

For the TEM studies, a morphogenic callus of Brachypodium was fixed in 2.5% glutaraldehyde in a 0.1 M sodium phosphate buffer (PB; pH 7.4) at 4 °C for 24 h. After washing in phosphate buffered saline (PBS, 5 times, 30 min each), the material was postfixed in 1% osmium tetroxide in a 0.1 M PB (4 °C, 24 h), rinsed with the same buffer and dehydrated in a graded concentration series of ethanol (30%, 50%, 70%, 90%, 96%, and 100%, each for 15 min) and acetone (two times, 15 min each) followed by infiltration in mixtures of acetone and Epon 812 resin (3:1, 1:1, and 1:3) (Polysciences, Eppelheim, Germany). Then, the material was embedded in Epon 812 resin and polymerized into resin blocks at 60 °C for 48 h. Ultra-thin (70 nm) sections were cut with a diamond knife on an Ultracut UCT25 (Leica, Wetzlar, Germany) ultramicrotome. After contrast staining with uranyl acetate and lead citrate, the sections were examined using a H500 transmission electron microscope (Hitachi, Tokyo, Japan).

### 4.4. TUNEL Assay 

The TUNEL methodology was according to Kwasniewska et al. [72]. The callus tissue was fixed with 4% paraformaldehyde for 1 h at room temperature on the seventh day of cultivation and then washed 3× for 5 min in PBS. The squashed slides with nuclei were prepared in PBS and were frozen at −70 °C and air dried at room temperature. Next, the slides were incubated in a permeabilization solution (0.1% Triton X-100 in 0.1% sodium citrate) for 2 min at 4 °C, and then rinsed with PBS. The positive control was prepared by adding 50 µL of a DNase solution (250 µg/mL) to a slide that had been prepared from the control material for 30 min at 37 °C in a humid chamber. After DNase treatment, the slides were rinsed twice with PBS. DNA fragment labelling was carried out using the TUNEL reaction mixture (In Situ Cell Death Detection Kit, Fluorescein, Sigma-Aldrich, St. Louis, MO, USA). A 50 µL measure of the TUNEL reaction mixture (enzyme: fluorescein-labelled nucleotides, 1:9 ratio, *v*/*v*) was added to the preparation and incubated in a humid chamber in the dark for 1 h at 37 °C. For the negative control, only 50 µL of the reaction mixture without terminal transferase was used. The slides were rinsed 3× with PBS.

The slides were stained with DAPI (2 µg/mL) and mounted in Vectashield (Vector Laboratories, Peterborough, United Kingdom). Preparations were examined with an Axio Imager Z2 wide-field fluorescence microscope equipped with an AxioCam Mrm monochromatic camera and the appropriate sets of filters (Zeiss). The frequency estimation of the labelled nuclei was based on an analysis of at least 500 cells from two slides for each treatment and period/duration of culturing. 

### 4.5. Gene Expression Analysis

Trizol (Invitrogen, Carlsbad, CA, USA) plus RNeasy Mini Kit (Qiagen, Hilden, Germany) was used to isolate total RNA from the ZEs [73] and 200 explants were used for each biological replicate. The concentration and quality of the isolated RNA was evaluated using an ND-1000 NanoDrop spectrophotometer (Waltham, MA, USA). First-strand cDNA was produced using a Maxima H Minus First Strand cDNA Synthesis Kit with dsDNase (Thermo Scientific, Waltham, MA, USA). The product of the reverse transcription was diluted with water at a 4:1 ratio and 2 µL of this solution was used for the Real Time RT-qPCR reactions using a LightCycler^®^ 480 SYBR Green I Master in a LightCycler^®^ 480 Real-Time PCR System (Roche, Basel, Switzerland). The primers that were relevant to the genes that were studied are listed in Table 1. The relative RNA levels were calculated and normalized to an internal control, the AK437296 gene encoding ubiquitin. This control gene exhibited a constant expression pattern (*C*_t_ = 17 ± 1) in all of the tissue samples that were analyzed. The plant tissues for the real-time RT-qPCR analysis were produced in three biological repetitions and two technical replicates of each repetition were carried out. The relative expression level was calculated using 2^−dd*C*t^, where dd*C*_t_ represents d*C*_t_^reference condition^ − d*C*_t_^compared condition^.

### 4.6. Immunocytochemistry

The fixation and embedding procedure was prepared according to Betekhtin et al. [2]. Sections were incubated in a blocking buffer containing 2% (*v*/*v*) foetal calf serum (FCS) and 2% (*w*/*v*) bovine serum albumin (BSA) in PBS (pH 7.2) for 30 min at room temperature. Next, they were incubated with specific primary monoclonal antibodies (Table 2), diluted at a ratio of 1:20 in a blocking buffer (room temperature, minimum 1.5 h), rinsed with the blocking buffer 3 × 10 min and then incubated at room temperature for at least 1.5 h with the secondary antibody (Alexa Fluor 488 goat anti-rat IgG, Jackson Immuno-Research Laboratories, West Grove, PA, USA), which was diluted at a ratio of 1:100 in the blocking buffer as above. After washing with the blocking buffer and PBS (3 × 10 min each), the sections were stained with 0.01% (*w*/*v*) calcofluor (Sigma-Aldrich, St. Louis, MO, USA) in PBS for 5 min, then the slides were thoroughly rinsed with PBS and sterile distilled water (3 × 10 min each). Drained slides were mounted in a Fluoromount (Sigma-Aldrich) antifade medium. Negative controls were performed for each antibody that was used by omitting the primary antibodies. The greenish background on the figures is presence as the results of autofluorescence because the stronger exposure time was applied. This procedure because of the weaker fluorescence signals from antibodies in experiments with 5-azaC compare to control cells was done. All images were taken using an Axio Imager Z2 epifluorescence microscope equipped with an AxioCam Mrm monochromatic camera (Zeiss) with the narrow-band filters for AlexaFluor 488 and DAPI. For general histology, the sections were stained with a 0.05% (*w*/*v*) toluidine blue O for 5 min.

## 5. Conclusions

We have demonstrated that Brachypodium tissue culture can be a useful model system to reveal the influence of 5-azaC during the formation of EM in grasses as well as for studying cell death in monocotyledonous plants. Future work including analyzes of SE induction in Brachypodium of the genes encoding for DNA methylases/demethylases should be considered. 

## Figures and Tables

**Table 1:** Oligonucleotide primers used for RT-PCR reaction with the relevant description of the genes.

Genes	Description of the Genes	Primer Sequence (5′–3′)
AK437296	ubiquitine	pF–GAGGGTGGACTCCTTTTGGA
		pR–TCCACACTCCACTTGGTGCT
Bradi3g01470.1	aminoacylase activity, catalase activity	pF–TTGTGAAGAGGTTCGCGGATGC
		pR–TCCCACACGACTTATCACACTGG
Bradi3g35620.1	putative glutathione S-transferase	pF–TTTCCATTGCTGAGCACAAGAGC
		pR–GGGACTTGACCAAATGGATTGCG
Bradi5g09650.1	thioredoxin peroxidase activity	pF–GAACCCTTCAGGCCCTGCAATATG
		pR–AACCTGCTGGGCAAACCTCATC
Bradi2g18877.1	hexokinase	pF–AATGACACGGTAGGCGAGGATG
		pR–GACTTTCATATCGAGACCCTGACG
Bradi2g18290.1	single-stranded DNA specific 5′–3′ exodeoxyribonuclease activity	pF–AGGCACCTTGTGAAGCAGAAGC
		pR–TCTGATGCGACAGCATACACCTTG
Bradi1g75270.1	pheophorbide oxygenase (PAO, ACD1)	pF–ACCGTCCTTTCAAAGCGTGAGATG
		pR–CGCTCCTTTGCAAGACGAACAC
Bradi1g60762.1	metacaspase involved in regulation of apoptosis	pF–ACTGCATCCTCATCCTCACAGAG
		pR–AGCCAGCAGATTCTCCTTCGTC
Bradi1g60756.1	metacaspase involved in regulation of apoptosis	pF–ACTGCATCCTCACCCTTACACC
		pR–AGAAGTGGAACACCAGGGAGTC
Bradi1g05570.1	BAX inhibitor related	pF–ACGCCATCGTCCTGATGTTGTTC
		pR–TGAGGAAGGCCGAGAAGATGAGC
Bradi1g02697.1	XP-G/RAD2 DNA repair endonuclease family	pF–AGGGTTTGACGAAGCTGCTG
		pR–TCCTTTCCTTCCTACCACAACCAG
Bradi3g01470.2	aminoacylase activity, catalase activity	pF–AGGTGATGGACCCAGATGAG
		pR–GAAGTTGTCCACGTTTCGGT
Bradi3g29270.1	protein executer 1	pF–GTTGGTGGCAACAGGAAACT
		pR–GAATTCGGCTGAAGTGGGTA

**Table 2:** Antibodies used for immunocytochemistry, the epitopes they recognize, and relevant references.

Antibody	Epitope	References
	Arabinogalactan proteins (AGPs)	
JIM8	Arabinogalactan	[74]
JIM13	(beta)GlcA1→3(alpha)GalA1→2Rha	[75]
	**Extensins**	
LM1	Extensin	[76]
JIM11	Extensin	[77]
JIM12	Extensin	[77]
